# Early postoperative oral feeding shortens first time of bowel evacuation and prevents long term hospital stay in patients undergoing elective small intestine anastomosis 

**Published:** 2019

**Authors:** Behzad Nematihonar, Akram Yazdani, Rofeideh Falahinejadghajari, Alireza Mirkheshti

**Affiliations:** 1 *Imam Hossein Hospital, Shahid Beheshti University of Medical Sciences, Tehran, Iran*; 2 *Biostatistic Department, Tehran University of Medical Sciences, Tehran, Iran*

**Keywords:** Intestine anastomosis, Bowel evacuation, Oral feeding, Hospitalization

## Abstract

**Aim:**

This study was conducted to compare outcome of early oral feeding (EOF) versus traditional oral feeding (TOF) in patients undergoing elective small intestine anastomosis.

**Background:**

Appropriate nutritional support after major surgeries is a real medical concern. As traditional surgical techniques have been replaced by novel methods, postoperative care should be revised as well. Early postoperative oral feeding was studied in trauma and burn. However, there are few trials among patients after major surgeries.

**Methods:**

This randomized single-blinded controlled trial was performed on 108 patients who had small intestine anastomosis at Imam Hossein Medical Centre in 2012. The patients were randomly assigned to schedule EOF (with starting oral feeding on the first day after surgery and complete return of the Gag reflex) or TOF (with delaying oral feeding till first passage of flatus and bowel movement). We compared overall prevalence of postoperative complication, length of hospital stay and outcome of surgery in two groups.

**Results:**

The time of the first passage of stool was shorter in EOF group than in TOF group (3.2 ± 0.59 days versus 3.6 ± 0.66 days (p= 0.006). The mean length of hospital stay in EOF group was also shorter than in TOF group (3.8 ± 1.06 days versus 6.3 ± 1.0 days, p= 0.001). The length of hospital stay shorter than 4 days was found in 75.9% of patients in EOF group and 11.1% of those patients in TOF group (p < 0.001).

**Conclusion:**

The use of EOF in patients undergoing small intestine anastomosis can shorten time of the first passage of stool as well as reduce length of hospital stay.

## Introduction

 Appropriate nutrition following major surgeries is a main purpose of postoperative supportive care. There are concerns about initiation of oral feeding in the immediate postoperative period. On the other hand, there are increasing number of patients who need abdominal surgeries. As an illustration Pourhoseingholi *et al.*, revealed that years of life lost due to colorectal cancers have an increasing trend in Iran (1). Many of these patients need surgeries and anastomosis. Due to probable ileus after abdominal operations, beginning early oral feeding may be avoided and thus nasogastric decompression is preferred (2). After abdominal surgeries, initiation oral nutrition depends on passage of flatus or bowel movements as an indicator of ileus resolution (3). It has been shown that early enteral feeding in these patients may have some potential advantages, reduces postoperative morbidities, mortality and septic events when compared to parenteral nutrition (4,5). By advancing laparoscopic abdominal surgery, the safety and tolerability of early oral nutrition has been more emphasized. However, the major controversies have been remained which related to the safety of early oral nutrition in comparison with traditional delayed nutrition following small bowel resection and anastomosis. In fact, although the beneficial consequences of early feeding have been shown in patients with trauma or burn (6-7), a few studies have focused its safety among patients undergoing gastrointestinal surgeries. Hence, this study aimed to compare the outcome of early oral feeding compared to traditional feeding in patients undergoing elective small intestine anastomosis. 

## Methods

**Study Participants**

This randomized single-blinded controlled trial was performed on 108 patients who undergoing small intestine anastomosis using Gambee protocol at Imam Hossein Medical Centre, Tehran in 2012, after the approval by ethical committee of Shahid Beheshti University of Medical Sciences. Those patients aged less than 18 years or older than 75 years, or with the history of laparoscopic surgery, severe cardiovascular disorders (heart failure with cillip class III to IV), immunosuppressive conditions, history of recent corticosteroid use, pregnancy, bowel obstruction proximal to the site of anastomosis, shock, low serum albumin level (less than 3.0 mg/dl), electrolyte imbalance, or severe anemia (serum hematocrit less than 15%) were all excluded from the study. The included patients were randomly assigned to two groups receiving early oral feeding (with starting oral feeding on the first day after surgery and complete return of the Gag reflex) or traditional feeding (with delaying oral feeding till first passage of flatus and bowel movement). After operation, nasogastric tube was quickly removed. In early oral feeding group, the nutrition began by taking a clear liquid on the first day after surgery and was turn into normal diet if the regimen tolerated within 24-48 hours after. In control group, the traditional method routinely began after ileus resolution indicated by passage of flatus or stool. The nutritional regimens were similar in both intervention groups except for diabetic patients that received diabetic regimen. The patients ‘characteristics were assessed using a self-administered questionnaire designed by the researchers and under supervision of an epidemiologist and a surgeon. After designing, the questionnaire was validated and its reliability was tested by assessing the Chronbach’s alpha that was shown to be 0.84. The questionnaire consisted of three main parts including 1) baseline characteristics and clinical history including demographics, underlying disorders, history of abdominal surgeries, history of chemotherapy or radiotherapy, and also medications, 2) disease-related factors such as patients’ complaints, type of operation, biochemical markers, vital signs on admission, and occurring intraoperative or postoperative shock, and 3) postoperative outcome central temperature 48 hours after beginning nutrition, early and late postoperative complications, time to first passage of flatus or stool, and time of discharge from hospital .The one who filled the questionnaire was not aware of which group were patients assigned in to. The early outcome was defined as the presence of at least one of these complications: nausea, vomiting, pneumonia, or sinusitis. Also, late outcome was referred to the presence of at least one of these complications: peritonitis, intra-abdominal abscess, sepsis, leakage from anastomosis site, intestinal fistula, or death. All patients were hospitalized for at least one week. The criteria for discharging the patients were lack of nausea and vomiting, abdominal distension, oral feeding tolerability, partial recovery, the absence of fever and normal vital signs. 

**Statistical Analysis**

In order to detect a mean difference of 1 day in the postoperative length of hospital stay, a sample size of 54 patients for each group was calculated, with an alpha of 0.05, an expected standard deviation within the groups of 1.7 days, and a power of 0.86. 

The results were expressed as mean ± standard deviation (SD) for normal variables, med±IQR for non-normal variables and they were summarized by frequency (percentage) for categorical variables. Continuous variables were compared using t test or Mann-Whitney U test were used to analyze nonparametric data. The assumption of normality for continuous variables was investigated using the Kolmogorov-Smirnov test, and if the assumption of the normality was made, T-test was used to compare the mean of the two groups. If the assumption of the normality was not satisfied, non-parametric test replaces T-test in comparison mean between two independent groups, the Mann-Whitney test was used. Categorical variables were, on the other hand, compared using Chi-square or Fisher's exact test. Significance was considered for values of P < 0.05.

For the statistical analysis, the statistical software SPSS version 16.0 for windows (SPSS Inc., Chicago, IL) was used. P-values of < 0.05 were considered statistically significant.

## Results

In total, 108 patients were randomly assigned to receive early oral feeding (EOF, n = 54) or traditional oral feeding (TOF, n = 54). The two groups were similar in terms of mean age (50.58 ± 18.20 years in TOF versus 46.10 ± 13.92 years in EOF, p = 0.41). As shown in table 1, there was no difference between the two groups in underlying disorders and even cause of surgery, history of radiotherapy or chemotherapy, oral medications. In TOF group, the most reasons for surgical anastomosis included strangulated hernia (24.5%), gastrointestinal cancer (22.6%), abdominal trauma (22.6%), gastric outlet obstruction (13.2%), intestinal obstruction (5.7%), closing ileostomy (7.6%), and gastrointestinal bleeding (3.8%); while in EOF group, the main reasons were strangulated hernia (29.6%), abdominal trauma (25.9%), cancer (18.5%), intestinal obstruction 14.8%), and gastric outlet obstruction (11.1%). With respect to the type of surgery, in TOF group, gastrointestinal anastomosis was performed in 25.9%, intestino-intestinal anastomosis in 61.1%, and colon anastomosis in 13.0%, while these types of anastomoses in EOF group were found in 20.4%, 68.5%, and 11.1%, respectively with no difference (p = 0.72).

Comparing biochemical markers between two groups (table 2) showed that except for serum levels of hemoglobin that was lower in TOF than in EOF groups, no difference was revealed in other markers between groups. Also, comparing hemodynamic parameters showed higher mean systolic blood pressure in TOF compared to EOF group, but no difference was found in diastolic blood pressure, pulse rate, and central body temperature between the groups (table 3).

The outcome of surgery is described in table 4 The mean time for beginning oral feeding in TOF group was 4.3 ± 0.87 days, while feeding was begun a day after surgery in EOF group (p < 0.001). The mean body temperature on beginning of nutrition was lower in EOF than TOF group (p = 0.0003), whereas no difference was observed in body temperature 48 hours after that (p = 0.36). The time to first passage of flatus in EOF and TOF groups was 2.1 ± 0.75 days and 2.2 ± 0.68 days, respectively with no difference (p = 0.55). However, the time to first passage of stool was shorter in EOF group than in TOF group (3.2 ± 0.59 days versus 3.6 ± 0.66 days (p = 0.006). The mean length of hospital stay in EOF group was also shorter than in TOF group (3.8 ± 1.06 days versus 6.3 ± 1.0 days, p = 0.001). The length of hospital stays shorter than 4 days was found in 75.9% of patients in EOF group and 11.1% of those patients in TOF group (p < 0.001). The overall prevalence of postoperative complication was 19.5% in EOF group and 22.2% in TOF group with no difference. In this regard, the prevalence of nausea and vomiting in EOF group was 16.7% and 1.85% and in TOF group was 20.35% and 1.85%, respectively (p = 0.88).

**Table 1:** Frequency distribution and statistics by gender, age and underlying disease of the patients under study.

**Variable**	**Group**		**P-value**
	**EOF**	**Traditional**	
Age(year)	64.10 ± 13.9	50.58 ± 18.20	0.412
Male	26 (48%)	24 (44%)	0.943
Female	28 (51%)	30 (55%)	
Underlying diseases			
No history	35 (64.8)	39 (72.2)	0.254
Diabetes mellitus	5 (9.3)	3 (5.6%)	
Hypertension	8 (14.8)	3 (5.6%)	
Cancer	2 (3.7%)	4 (7.4%)	
Thyroid diseases	2 (3.7%)	0 (0%)	

EOF: Early oral feeding. The reported numbers are the number and percentage of patients in each group

**Table 2:** Comparison of the two groups in terms of biochemical marker variables

**Variable**	**Traditional group**	**EOF group**	**P-value**
Fasting blood sugar	95.22±0.65	99.14±0.75	0.061
Urea	68.20±7.3	72.23±5.6	0.272
Albumin	3.7±0.47	3.9±0.55	0.098
Globulin	3.0±0.8	2.9±0.9	0.900
Albumin/Globulin ratio	1.3±0.44	1.6±0.96	0.172
Total protein	6.7±0.8	6.8±1.1	0.273
Hemoglobin	11.3±1.7	11.8±1.6	0.013
Creatinine	0.89±0.33	0.9±0.6	0.772
Na	139.3±0.5	139.15±9.2	0.283
k	4.0±0.25	4.1±0.31	0.924

FBS(mg/dl), Urea(mg/dl), Globulin(g/dl), albumin(g/dl), Total protein(g/dl), Hb (g/dl); Creatinine(mg/dl); Na(mEq/L). K(mEq/L) EOF: Early oral feeding

**Table 3:** Comparison of the two groups in terms of hemodynamic parameters

**Variable**	**Traditional group**	**EOF group**	**P Value**
med±IQR			
Systolic Blood Pressure	115±10	105±14	0.004
Diastolic Blood Pressure	77±9	79±8	0.501
Mean±SD			
Heart Rate	88.9±8.3	88.1±0.9	0.991
Core Temperature at the beginning)	37.2±0.5	37.3±0.5	0.492

BP (mmhg), Heart Rate (N/min), Temprature (°C), EOF: Early oral feeding

**Table 4:** Comparison of two groups regard to questionnaire

**Variable**	**Traditional group**	**EOF group**	**P Value**
Day of beginning oral feeding	4.3±0.87	1.0±0.0	˂0.001
Body temperature at the beginning of nutrition	37.7±0.5	37.2±0.36	˂0.001
Body temperature after 48 hours	37.1±0.5	37.1±0.25	0.362
First passage of flatus (day)	2.2±0.68	2.1±0.75	0.553
First passage of stool(day)	3.6±0.66	3.2±0.59	0.006
Hospital stay (day)	6.3±1.0	3.8±1.06	˂0.001

EOF: Early oral feeding

## Discussion

According to our findings, considering early oral feeding in patients who undergoing intestinal anastomosis improve clinical consequences of surgery with respect to preserve glucose and hemoglobin levels as well as preventing systolic blood pressure rising. In fact, regardless of the time of first passage of flatus and stool, early oral feeding results in favorable outcome in the patients after intestinal anastomosis. The main purpose of the immediate starting oral nutrition and early nutritional support in these patients is to prevent catabolic effects of disease as well as inhibit disease or surgery-related damages. Thus, early oral feeding in the patients can be accompanied with these clinical and hemodynamic preservations and can support clinical evidences on beneficial effects of early oral feeding in patients. According to our results, almost all patients in EOF group tolerated early oral feeding without major and serious complications. More interestingly, the time to first defecation were sooner in the EOF group than the traditional group. Also, because of more appropriately controlling hemodynamic status and setting biochemical stability in EOF group than in TOF group, the former group needed shorter length of hospital stay. Our findings were compatible with the results of early oral feeding in patients have undergone colorectal anastomosis by Nematihonar *et al.* (8). They demonstrated that early feeding in patients with colorectal anastomosis leads to shorter bowel sounds auscultation after surgery and shorter time for resolution of ileus after surgery. Reduction of the first flatus and feces, shorter overall stay, and greater patient satisfaction with the treatment process were all significant. In a meta-analysis by Osland *et al.* (9) and review of 15 studies comparing early versus traditional oral feeding, it was shown a statistically significant reduction in relative odds of total postoperative complications in patients receiving EOF. Also, no effect of early feeding was seen with relation to anastomotic dehiscence, mortality, days to passage of flatus, first bowel motion, or reduced length of stay; however, the direction of clinical outcomes favored early feeding. Similar to our study, Thapa *et al.* (10) in 2011 showed that EOF in patient undergoing major gastrointestinal surgery resulted in early return of bowel movement, decreased ICU and hospital stay with a significant reduction in postoperative cost. Böhm *et al*. (11) prospectively analyzed patients and showed 60% tolerating EOF on postoperative day 3, 74% on postoperative day 4, and 88% on postoperative day 5. Tong *et al*. (12) reported that 73% of the patients tolerated EOF without sequelae. Also, comparable to our finding with respect to shorter hospital stay following EOF regimen, Tong *et al.* (12) and Hjort *et al*. (13) revealed that the medium hospital stay was 2 days after EOF regimen while it was 8 days after conventional feeding. Reissman *et al*. (14) reported that early feeding did not affect the length of ileus and did not significantly shorten the length of hospitalization. In fact, it seems that EOF regimen early after gastrointestinal surgery is safe, well tolerated, and can improve postoperative gastrointestinal motility, and therefore can play an important role in enhanced recovery and outcome (15). In total, it is now believed that although EOF regimen might increase the risk for anastomotic leakage, the pointed complication was not reported in our study. In fact, some strong evidence are even available in the strengthening effect of adequate EOF regimen on intestinal anastomoses without leading anastomotic complications (16). It has been well demonstrated that early feeding can reverse the mucosal atrophy induced by starvation and increases anastomotic collagen deposition and strength (17-19), heal surgical wound (20-21), and even improve patients' sense of well-being (22). In a systematic review and meta-analysis, Willcutts *et al.* demonstrated that early postoperative oral feeding as compared to traditional (or late) timing is associated with shorter hospital length of stay and is not associated with an increase in clinically relevant complications (23). These results were confirmed by another meta-analysis which was conducted by Xiaoping Liu, *et al*. The result of this meta-analysis showed that EOF after gastric cancer surgery seems feasible and safe, even started at the day of surgery irrespective of the extent of the gastric resection and the type of surgery (24).

This study revealed results of EOF in patient with small intestine problems and anastomosis. To our best knowledge, there is less data about the small intestine surgeries among different types of abdominal surgeries. The results may clear up the doubt about controversies which prevent physicians responsible for postoperative nutrition, from initiation feeding in immediate period after surgery. Despite limitations such as, not collecting data about long term complication and absence of double-blind trial, the authors believe that the results would be beneficial in clinical practice.

We suggest further studies at the level of cellular technology and metabolomics to be conducted to improve postoperative care. 

Finally, it can be concluded that considering EOF rather than TOF regimen does not lead to anastomotic leakage or surgery-related complications in patients undergoing intestinal anastomosis. In fact, the use of EOF in these patients can reserve hemodynamic status, prevent systolic blood pressure raise, shorten time of first passage of stool as well as reduce length of hospital stay.
